# Enhanced targeted integration mediated by translocated I-SceI during the *Agrobacterium* mediated transformation of yeast

**DOI:** 10.1038/srep08345

**Published:** 2015-02-09

**Authors:** Martijn Rolloos, Paul J. J. Hooykaas, Bert J. van der Zaal

**Affiliations:** 1 Department of Molecular and Developmental Genetics, Institute of Biology Leiden, Leiden, Sylviusweg 72, 2333 BE, The Netherlands

## Abstract

*Agrobacterium* mediated transformation (AMT) has been embraced by biotechnologists as the technology of choice to introduce or alter genetic traits of plants. However, in plants it is virtually impossible to predetermine the integration site of the transferred T-strand unless one is able to generate a double stranded break (DSB) in the DNA at the site of interest. In this study, we used the model organism *Saccharomyces cerevisiae* to investigate whether the *Agrobacterium* mediated translocation of site-specific endonucleases via the type IV secretion system (T4SS), concomitantly with T-DNA transfer is possible and whether this can improve the gene targeting efficiency. In addition to that, the effect of different chromatin states on targeted integration, was investigated. It was found that *Agrobacterium* mediated translocation of the homing endonuclease I-SceI has a positive effect on the integration of T-DNA via the homologous repair (HR) pathway. Furthermore, we obtained evidence that nucleosome removal has a positive effect on I-SceI facilitated T-DNA integration by HR. Reversely; inducing nucleosome formation at the site of integration removes the positive effect of translocated I-SceI on T-DNA integration.

A*grobacterium tumefaciens* is a soil born plant pathogen well known for its ability to genetically alter the cells of the dicotyledonous plants that form its main natural host. These genetically modified plant cells are inclined to produce amino acid derivatives, which can be utilized by *Agrobacterium* as a carbon and nitrogen source. In addition to this, plant tissue infected by *Agrobacterium* shows excessive cell proliferation resulting in tumorous outgrowths. Although for agriculturists these are the symptoms of crown gall disease, for biotechnologists the *Agrobacterium* mediated transformation (AMT) of plants has formed the foundation for new technologies leading the way to crop improvement and the production of medicines or biofuels by plants. The precursor of the DNA that is integrated into the plant genome is located on a tumor-inducing (Ti) plasmid flanked by two imperfect direct 25 bp repeats; the left and right border sequences^1^. This transfer-DNA (T-DNA) is processed by the *Agrobacterium* virulence protein VirD2 which induces a nick at the lower strand of the right and left border sequence^2^,^3^. A single stranded section of the T-DNA with VirD2 covalently attached to the 5′ end, the T-strand, is released and targeted to a type IV secretion system (T4SS) which injects the T-strand into the host cell^4^. There are two models for the integration of T-DNA in plants, one involves the free 3′-end of the T-strand to find microhomologies in the genomic DNA of the host cells. After the free 3′-end anneals to the genomic DNA, this would then prime the formation of the complementary strand^5^. Later an alternative route for T-DNA integration was suggested where double stranded DNA is formed from the T-strands prior to opportunistic integration in genomic DSBs^6^,^7^,^8^. Probably host cell primases can synthesize de-novo RNA primers complementary to the T-strand, thereby facilitating the synthesis of a secondary T-strand prior to integration^9^.

T-DNA tends to integrate randomly into the genome of the recipient cell in respect to predicted transcriptional activity based on the DNA methylation patterns^10^. Inherent to the randomness of T-DNA integration is the risk of unintended gene disruption at the integration site or deviating expression levels of the introduced transgene caused by different positional effects at integration sites. To address these concerns related to AMT, it would be highly desirable to utilize the homologous recombination (HR) system of plants to replace a predetermined genomic locus with an incoming T-strand that comprises a strong homology to that locus. Unfortunately, HR-mediated integration of T-DNA in plants is a rare event, estimated to occur only once per 10^4^ to 10^5^ integration events^11^,^12^.

A breakthrough regarding the targeted integration of T-DNA via HR was the finding that this is facilitated by the formation of DNA double strand breaks (DSBs) at the target locus^7^,^8^,^13^. With the development of highly versatile site specific artificial nucleases, it is now theoretically possible to generate a DSB at any particular genomic position of interest^14^ and a major bottleneck for site-directed mutagenesis has thus disappeared. However, without directly selectable gain of function mutations after gene targeting, finding the few HR-mediated gene targeting events remains challenging. Negative selection against randomly integrated T-DNA copies can be instrumental for the recovery of rare gene targeting events in rice^15^, but this seems not to be generically applicable in other plant species. Other studies towards enhancing gene targeting have explored down regulation of enzymes involved in the NHEJ pathway which was very efficient in yeast and fungi^16^ but also this approach has variable efficiency in plants^17^,^18^.

Altogether, despite the abundant use of AMT for plant transformation, reports about its successful application for gene targeting are still scarce and a generally applicable strategy is lacking. Additionally, obvious parameters likely to be important for successful application of AMT for gene targeting have still been insufficiently explored. The first parameter that is subject of the present study concerns the chromatin state of the target locus. It has been demonstrated that the effect of site specific artificial transcription factors very much depends on their access to DNA^19^,^20^. As a more open chromatin structure might allow for better access of site specific nucleases as well, our experiments have addressed the possibility of nuclease access of the chromosomes for different states of chromatin condensation. The second parameter that is addressed in this manuscript was related to the delivery of the required site specific nucleases to the recipient cells. In the present study, different approaches were explored to deliver nuclease proteins to the recipient cell via *Agrobacterium* mediated translocation concomitant with T-strand delivery as will be explained below.

*Agrobacterium* effector proteins are targeted for translocation to the recipient host cell because of a series of positively charged amino acids at their C terminal end that act as a T4SS translocation signal^21^. An illustrative example of this is the C terminal end of the virulence protein VirF (VirF^CT^). By making translational fusions with VirF^CT^ a number of DNA modifying proteins have been shown to translocate to the host cells, like the recombinase Cre^21^,^22^. Recently, it was demonstrated that complex fusion proteins consisting of a nuclear localization signal (NLS) for nuclear targeting, the *Agrobacterium* relaxase VirD2, I-SceI for the induction of double stranded breaks and a VirF^CT^ moiety to allow the translocation of this protein complex to the host cell can be translocated to recipient plant cells^23^.These experiments were performed by means of assaying simultaneous transfer of T-strands coupled to such fusion proteins. The occurrence of footprints within the I-SceI recognition site in the host genome proved that the I-SceI moiety retained its nuclease activity after *Agrobacterium* mediated translocation. Hence, T-strands can be equipped with covalently attached modified VirD2 proteins, combining the relaxase activity of VirD2 with enzymatic activities of choice^24^.

A possibility to achieve gene targeting via AMT by introducing particles consisting of a T-strand with target site homology, covalently linked with a nuclease moiety that creates the required DSB to trigger an HR-mediated gene targeting event is well worth exploring. However, AMT offers opportunities to deliver the T-strand and the nuclease via the T4SS also without covalent linkage of the nuclease to a T-strand. In these cases the T-strand passes to the recipient cell piloted by an unmodified VirD2 protein bound at its 5′end, while the nuclease of interest is produced in the same *Agrobacterium* cell and is transported via the T4SS by means of a VirF^CT^ fusion.

Previously, it was shown that AMT of *Saccharomyces cerevisiae* (budding yeast) can be used as a model system to address the roles of HR and NHEJ pathways in T-DNA integration^16^,^25^. Readily available mutants, time-efficient transformation protocols for the introduction of genomic target loci, as well as convenient transformant screening and subsequent analysis contributes to making the AMT of yeast a valuable asset. Of special interest to this study are the well characterized promoter regions of yeast genes involved in the galactose pathway that, depending on the carbon source, can have open or closed chromatin states^26^. These promoter sequences hence should allow for the *in vivo* modulation of the chromatin state during the AMT of yeast. In the present work, we used the yeast model to assess the possibilities for combined *Agrobacterium* mediated introduction of T-strands and the site-specific nuclease I-SceI to enhance gene targeting. In addition to this, the effects of the chromatin state for gene targeting via AMT were explored. These experiments were done in experimental set-ups conducive for transfer of T-strands covalently attached to NLS-VirD2-I-SceI-VirF^CT^ fusion proteins or, alternatively, for the transfer of T-strands covalently attached to an unaltered VirD2 molecule with a NLS-I-SceI-VirF^CT^ fusion protein translocated separately, albeit from single *Agrobacterium* cells.

As is shown in this manuscript, without introduced nucleases, the chromatin state of a target locus was of little importance for gene targeting frequencies. The *Agrobacterium* mediated transfer of a site specific nuclease protein during AMT resulted in higher transformation frequencies. This elevated efficiency of T-DNA integration, when transferring I-SceI fusion proteins with the T-strands, was however abolished when nucleosome formation was induced at the target locus.

## Methods

### The construction of the yeast target lines RSY12 pID2GU and RSY12 pIDU

For the amplification of the *UAS* typed promoter sequences from the yeast galactose pathway, genomic DNA from the *Saccharomyces cerevisiae* strain YPH250^27^ was used as a template. The PCR is performed using the primers listed in table 1. The PCR product from *UAS7* was digested with *Bam*HI and *Xho*I and cloned into pSKN*SgraI* which was also digested with *Bam*HI and *Xho*I. The resulting vector was named pOPSL3. The PCR product from *UAS10* was digested with *Spe*I and *BamH*I and cloned into pSKN*Sgra*I also digested with *Spe*I and *Bam*HI. The resulting vector was named pOPSL5. The cloning vector pSKN*Sgra*I has been described by Neuteboom *et al*^28^. After these initial cloning steps pOPSL5 was digested with *Bam*HI and *Spe*I. The resulting fragment containing the *UAS10* fragment was cloned into pOPSL3 digested with *Bgl*II and *Spe*I resulting in a new vector containing both upstream activating sequences; p2Gstart. This vector in turn was digested with *Spe*I and *Xho*I. A PCR fragment of *URA3* digested with *Spe*I and *Xho*I was cloned in between the upstream activating sequences resulting in p2GU. The primers used for the amplification of *URA3* are depicted in Table 1.

The shuttle vector pRS316^27^ was used as template for the PCR. From p2GU a *UAS10*-*URA3*-*UAS7* *Bam*HI fragment was excised and cloned into pINT^29^, a vector that integrates into the *PDC6* locus^30^ resulting in pID2GU. The intact *PDC6* locus contains a weakly expressed pyruvate decarboxylase (*PDC*) gene that is not required for full PDC activity in yeast cells^30^,^31^. To prepare the pINT vector for this cloning step it was digested with *Bam*HI and *Sph*I and treated with T4 DNA polymerase to create blunt ends recreating the *Bam*HI recognition site which was used to clone the *UAS10*-*URA3*-*UAS7* *Bam*HI fragment into. As a negative control, another target locus was required without the galactose inducible promoter sequences to rule out any down or upstream effects of the UAS nucleosome status on the neighboring *PDC6* locus. For this, the digested PCR product of *URA3* with *Xho*I and *Spe*I overhangs was cloned directly into pINT digested with *Sal*I and *Spe*I. The yeast strains RSY12 (*MAT****a**** leu2-3, 112 his3-11, 15 ura3D::HIS3*^32^) and YPH250 (*MATα, ura3-52, lys2-801, ade2-101, trp1-Δ1, his3- Δ 200, leu2- Δ 1*^27^) were transformed with pID2GU and pIDU by LiAc/ssDNA/PEG transformation^33^.

### The construction of the yeast target lines pID2G2SU and pID2G2LU

The plasmids pID2G2SU (pINT:*PDC6*:*UAS10*:*I-SceI RS*:*URA3*:*I-SceI RS*:*UAS7*:*PDC6*) and pID2G2LU (pINT:*PDC6*:*UAS10*:*lox*P:*URA3*:*lox*P:*UAS7*:*PDC6*) were constructed by the successive addition of two DNA fragments containing the enzyme recognition sites, directly upstream and downstream the *URA3* autotrophy marker gene of pID2GU (pINT:*PDC6*:*UAS10*:*URA3*:*UAS7*:*PDC6*). Pairs of complementary oligos depicted in Table 2 were annealed resulting in dsDNA fragments with ss overhangs compatible with pID2GU, successively digested with *Xho*I and *Spe*I.

The yeast strains RSY12^32^ and YPH250^27^ were transformed with pID2G2SU and pID2G2LU by LiAc/ssDNA/PEG transformation^33^.

### The construction of the binary and protein translocation vectors for *Agrobacterium*

Two types of vectors were used; binary vectors for the transfer of T-DNA and a set of vectors involved in protein translocation expressing chimeric DNA modifying proteins equipped with a translocation signal. For the construction of the binary vectors p14-2GKX (allowing for the translocation of T-DNA harbouring *UAS10*:*KanMX:UAS7*), and p14-PDC6KX (allowing for the translocation of T-DNA harbouring *PDC6*:*KanMX:PDC6*), the following steps were taken. A *KanMX* marker gene flanked with *Spe*I and *Xho*I restriction sites was generated by PCR using pSDM8000^16^ as DNA template. This fragment was cloned into p2GU also digested with *Spe*I and *Xho*I resulting in p2GKX. The fragment containing the *KanMX* marker gene flanked with the *UAS7* and *UAS10* sequence was cloned into the binary vector pSDM14^34^ as a *Kpn*I, *Sal*I fragment resulting into p14-2GKX. The PCR primers used for the amplification of *KanMX* are shown in table 3. To assemble a binary vector with two *PDC6* flanks homologous to the *PDC6* sequences as are present in pID2GU (pINT:*PDC6*:*UAS10*:*URA3*:*UAS7*:*PDC6*) and its derivatives, PCR was performed using the primers depicted in Table 3 using pINT as template. The resulting PCR product was digested with *Bgl*II and cloned into *Bam*HI digested pSDM14, resulting in p14-PDC. A *KanMX* marker gene with TEF1 promoter and terminator was obtained from pSDM8000 as a *BamH*I fragment and was cloned in p14-PDC digested with *BamH*I, resulting into p14-PDCKX. The binary vectors p14-2GKX and p14-PDC6KX were transferred to the *Agrobacterium* strain LBA1100 or LBA2556 by electroporation. LBA1100 has the C58C1 *Agrobacterium* chromosomal background and carries a disarmed octopine pTiB6 plasmid^35^. LBA2556 is a *virD2* null-deletion mutant, isogenic to LBA1100^36^.

For the construction of all the fusion protein translocation vectors describe here, pBFF was used as the vector backbone. This non-transmissable vector was derived from the broad host-range plasmid pRL662^37^ and was adjusted to enable the translocation of a diversity of fusion proteins^22^,^24^. Cloning ORFs as *Not*I fragments into the pBFF vector puts protein expression under control of the *virF* promoter sequence from the octopine Ti-plasmid. When properly allowing for translational fusions, produced proteins will contain an N-terminal FLAG-tag for immunodetection, a SV40 nuclear localization signal (NLS) to ensure its nuclear entry and a 37 aa C-terminal end of VirF allowing for T4SS mediated translocation^21^. The vector pBFF I-SceI and pBFF VirD2 I-SceI were generated by PCR-amplifying the I-SceI coding region (kind gift of Dr. Holger Puchta, University of Karlsruhe, Germany) and cloning it into the pBFF vector, thus supplying it with the features mentioned above.

### The construction of the yeast expression vectors

The yeast expression vectors were constructed by first cloning a DNA fragment encoding an ATG translational initiation codon followed by the *FLAG*::*NLS*::*virF^CT^* sequence into pRS425 ADH^38^ resulting into pRS425 ADH *FLAG*::*NLS*::*virF^CT^*. New ORFs were subsequently introduced as *Not*I fragments within the same site present directly upstream of *virF^CT^*. The vector pRS425 *FLAG*::*NLS*::*virD2*::*I-SceI*::*virF^CT^* was thus constructed by insertion of a *Not*I fragment from pBFF VirD2-I-SceI and pRS425 *FLAG*::*NLS*::*virD2*::*Cre*::*virF^CT^* by insertion of a *Not*I fragment from pBFF VirD2-CRE. The fusion proteins produced in *Agrobacterium* and yeast were derived from identical ORFs.

### Qualitative assay for enzymatic activity of DNA modifying fusion proteins in yeast

The pRS425ADH expression vectors encoding NLS-VirD2-I-SceI-VirF^CT^ or NLS-VirD2-CRE-VirF^CT^ were transferred to yeast cells carrying the different target loci by LiAc-transformation and transformants were selected for leucine autotrophy. After 3 or 4 days, 50 transformed yeast cells were removed from the selective plates and pooled in 500 μl of sterile 0.9% salt solution. The OD_600_ of this cell suspension was adjusted to 0.1 and diluted 100-fold. Subsequently, 100 μl of this cell suspension was replated on SD medium selective for leucine and uracil autotrophy, in order to maintain yeast cells carrying an expression vector and an intact target locus. The same aliquot of cells was plated on SD medium selective for leucine autotrophy but containing uracil as well as 5-FOA. The surviving colonies represented the yeast cells that carried the expression vector but lost the uracil selection marker gene.

For the AMT of yeast cells, we followed our earlier published method^39^ with some modifications. Briefly, *Agrobacterium* cells were precultured on LB medium with appropriate antibiotic selection. The transition from medium containing glucose to medium containing galactose has no impact on the growth rate of the *Agrobacterium* strains described here. For reliable results, the yeast strain YPH250 and RSY12 required acclimatization to galactose before and during the preculture step for sufficient growth on IM plates with galactose as the sole carbon source. Before mixing the *Agrobacterium* cell suspension with the yeast cells, the *Agrobacterium* cells were washed with induction medium (IM) without any carbon source. All cocultivations were performed for one week at 21°C, without any antibiotic or autotrophic selection in order to avoid any experimental biases. Whenever galactose induction of the yeast cells was applied, the IM used for preculturing the yeast strains as well as the induction plates were prepared with galactose instead of glucose.

## Results

### Experimental setup

**Figure 1:**
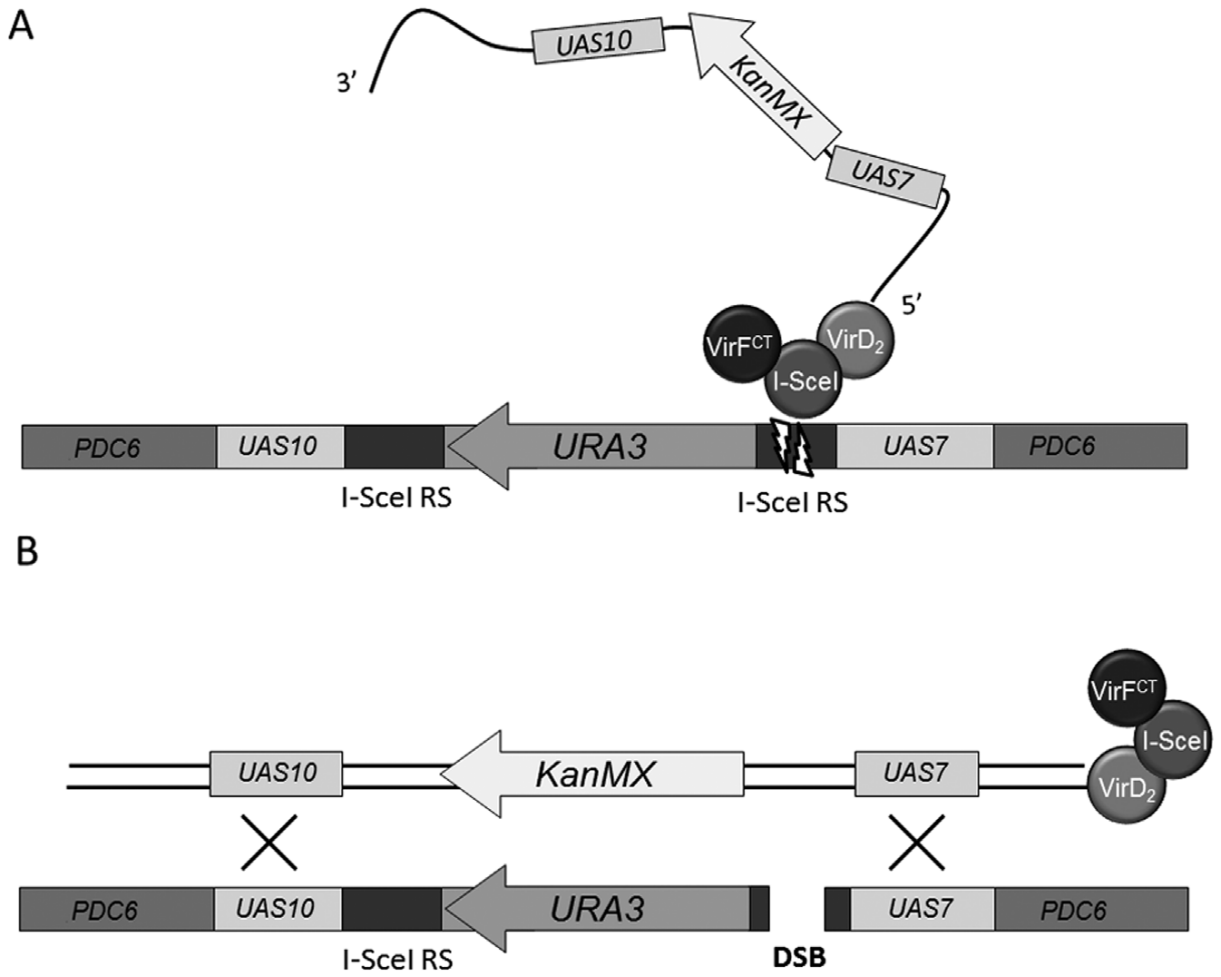
Experimental set-up for the study of T-DNA integration via HR by the I-SceI mediated DSB formation. (1A) The T-strand entering yeast cells is covalently attached to a fusion protein consisting of an FLAG-NLS module (not depicted in the figure), VirD2, I-SceI, and an *Agrobacterium* translocation signal derived from the VirF C-terminus. The I-SceI moiety induces the formation of a DSB at one or both the I-SceI recognition sites flanking the *URA3* gene, which triggers DNA recombination. (1B) Precise HR of the target locus with the incoming T-strand results in the exchange of the *URA3* autotrophy marker with the *KanMX* marker gene leading to G418 resistance as well as uracil auxotrophy. The regions on the T-strand homologous to the *UAS10* and *UAS7* sequences in the target locus are approximately 700 bp in length.

To get a deeper insight into the requirements of T-DNA integration by HR a versatile target locus was developed and introduced into the yeast genome. As can be seen in Figure 1, this target locus had a *URA3* marker gene flanked by two 18 bp I-SceI recognition sites. To enable the modulation of the nucleosome occupancy of this target locus, two different DNA sequences, normally present upstream of galactose inducible genes of the yeast galactose metabolism pathway, were cloned adjacent to the I-SceI sites. The same sequences, *UAS7* and *UAS10* were also introduced into a binary vector as part of the T-DNA sequence flanking an yeast KanMX marker which has no homology to the yeast genome. During *Agrobacterium* mediated transformation (AMT), a T-strand carrying these sequences will enter recipient yeast cells. With the *UAS7* and *UAS10* sequences, the T-strand can interact with the homologous chromosomal sequences at the target locus. Regarding the target locus, such an interaction can result in *URA3* for KanMX marker exchange by a two-sided HR event. As for the *UAS7* and *UAS10* sequences, their nucleosome occupancy is known to be reduced in the presence of galactose, and increased in the presence of glucose, resulting in increased and reduced expression of downstream ORFs, respectively. Detailed nuclease protection patterns for the sequences derived from yeast growing on galactose or glucose have become available^26^. The wide application of *UAS* sequences for carbon-source dependent gene expression provides compelling evidence that their characteristics, including the nucleosome occupancy, are portable traits.

In order to increase HR at the target locus, DSBs can be induced by I-SceI at the cognate restriction sites. We tested whether such DSBs can be induced efficiently by I-SceI translocated from Agrobacterium as a VirD2-I-SceI-VirF^CT^ fusion protein that is covalently attached to the translocated T-strand (Figure 1A). This protein contains an N-terminal FLAG tag followed by an SV40 nuclear localization signal (NLS) to ensure its nuclear entry as do the other fusion proteins used in this study. Since the full length VirD2 protein contains an active NLS sequence, an extra NLS is not really required^24^. For the I-SceI-VirF^CT^ protein, an active NLS is a prerequisite. Expression in *Agrobacterium* was mediated by the *virF* promoter.

The I-SceI moiety should induce one or more DSBs at the target locus, thereby enhancing the integration of T-DNA mediated by the HR pathway. Since the Holliday junctions involved in crossing over events during HR pathway require two dsDNA molecules, marker exchange is depicted as occurring between two double stranded DNA molecules (Figure 1B), thus as if the incoming T-strand has formed a complementary strand prior to integration. Incoming T-strands did not contain a nuclease target site.

**Figure 2:**
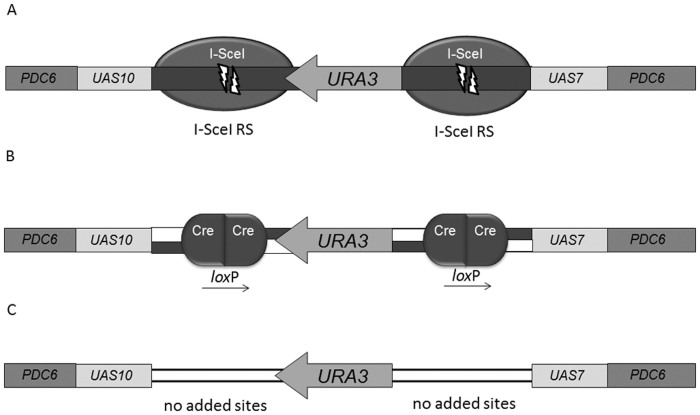
Target loci used to assay the effect of DSBs and nucleosome occupancy on the integration of T-DNA by HR. These target loci were all integrated at the *PDC6* locus, all containing sequence homology to the incoming T-strands by two different *UAS* type promoters derived from the yeast galactose pathway or by the flanking *PDC6* fragments. To remove nucleosomes from these promoter sequences, the yeast cells are grown in a medium with galactose as the sole carbon source. Growth in a glucose medium would induce the formation of nucleosomes on the *UAS* typed promoters. The central *URA3* marker is flanked by: (2A) recognition sites of the homing endonuclease I-SceI that catalyzes the formation of DSBs as a monomer, (2B) recognition sites for the recombinase Cre allowing for the removal of the *URA3* marker gene without inducing double stranded break formation, (2C) target locus without any added recognition sites but still provided with homology to the incoming T-strands.

The target locus depicted in Figure 1, as well as those shown in Figure 2, were integrated in the genomic *PDC6* locus. By performing the AMT of these yeast target lines on medium containing galactose or glucose, the respective removal or formation of nucleosomes can be induced and their influence on the integration efficiency can be assessed. For control experiments, alternative T-DNA regions were constructed, aimed to generate T-strands just representing the *KanMX* marker gene, thus without flanking *UAS* regions or to possess similarly sized flanking *PDC6* sequences instead of *UAS* regions (see Table 4).

AMT experiments with T-strands covalently attached to VirD2 containing fusion proteins, such as FLAG-NLS-VirD2-I-SceI-VirF^CT^, were performed using *Agrobacterium* strain LBA2556^36^ containing a Ti plasmid deleted for the *virD2* gene. For T-strand transfer combined with transfer of non-VirD2 containing proteins, such as FLAG-NLS-I-SceI-VirF^CT^, the isogenic strain LBA1100 was used. In this strain, a wild type VirD2 protein is still produced.

In addition to a target locus that is substrate for I-*Sce*I (Figure 2A), similarly designed target loci were prepared harboring recognition sites of the recombinase Cre (Figure 2B) and a negative control locus lacking any particular recognition sites (Figure 2C). A complete list of all gene constructs used for the experiments and their abbreviations is presented in Table 4.

### Enzyme activity of I-SceI and Cre fusion proteins

To test if the expressed fusions proteins were able to process the target loci described in Figure 2, an *in vivo* assay was designed. For this, the ORFs of the VirD2 fusion proteins were transferred to the yeast constitutive expression vector pRS425ADH equipped with a leucine autotrophy marker^38^. This resulted in pRS425ADH expressing NLS-VirD2-I-SceI-VirF^CT^ (pRS425 VirD2-I-SceI) and NLS-VirD2-Cre-VirF^CT^ (pRS425 VirD2-Cre) to serve as a positive control. These vectors were transferred to yeast cells carrying the corresponding target loci (derived from pID2G2SU and pID2G2LU respectively), or a negative control without any cognate recognition sites (derived from pID2GU). Fifty yeast colonies resulting from transformation with either of these expression vectors were pooled. An aliquot containing at least 1000 suspended yeast cells was plated on selective medium without leucine and uracil and on medium supplemented with 5-Fluoroorotic Acid (5-FOA) and uracil in order to determine the fraction of yeast cells that had lost the *URA3* marker, rendering them 5-FOA resistant. Although the described assay did not provide quantitative data on enzyme activity, it did show that the two VirD2 fusion proteins were able to process their cognate target loci but not the negative control target locus without recognition sites. The expression of I-SceI fusion proteins resulted in a complete loss of the *URA3* marker gene, Cre fusion proteins removed this marker in 88% of the cells.

### The effect of nucleosome occupancy on AMT transformation efficiency

**Figure 3:**
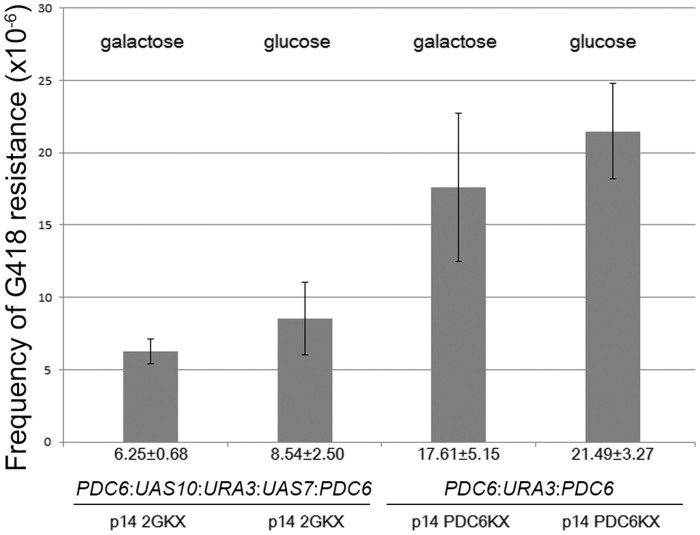
Effects of carbon source on AMT efficiency using yeast strain RSY12. Bar one and two represent RSY12 with the pID2GU target locus (*PDC6*:*UAS10*:*URA3*:*UAS7*:*PDC6*) cocultivated with LBA1100 harboring p14-2GKX. Bar three and four represent yeast cells with the pIDU target locus (*PDC6*:*URA3:PDC6*), lacking the *UAS* promoter sequences, cocultivated with LBA1100 harboring p14-PDC6KX (pSDM14:*rightborder*:*PDC6*:*KanMX:PDC6:leftborder*). Every bar represents the average of 10 independent experiments (n = 10). The transformation efficiency as is shown at the Y-axes should be read as ‘per million’ as is indicated with (×10^−6^). Error bars indicate the SEM. The hypothesis of equality could not be rejected with α set to 0.05 when performing a two-tailed heteroscedastic Student's T-test comparing “galactose” with “glucose”.

The yeast strain RSY12 harboring a pID2GU target locus (*PDC6*:*UAS10*:*URA3*:*UAS7*:*PDC6*) was cocultivated with an *Agrobacterium* strain translocating T-DNA from the binary vector p14-2GKX (*pSDM14:rightborder:UAS7: KanMX:UAS10:leftborder*). This T-DNA can integrate via HR at the *UAS* fragments present in the pID2GU target locus. Cocultivations on medium containing glucose or galactose, that should modulate the nucleosome occupancy of the *UAS* promoter sequences present at the target locus, resulted in similar transformation efficiencies. From these results it could be concluded that the presence or absence of nucleosomes at the *UAS* containing area of the target locus had no measurable effect on the efficiency of T-DNA integration (Figure 3, bar one and two). The same was observed for a negative control containing the pIDU reporter locus (*PDC6*:*URA3:PDC6*), that lacked the *UAS* promoter sequences, which was targeted by homologous T-DNAs derived from p14-PDC6KX which enables translocation of T-DNA with *PDC6*:*KanMX:PDC6* (Figure 3, bar three and four). From this result it could be concluded that there was also general effect of the different carbon sources on the transformation efficiencies.

### A comparison of two approaches to induce DSBs to affect T-DNA integration via HR

Using the yeast strain RSY12 harboring the target locus depicted in Figure 1, it was assessed whether it would be possible to obtain evidence for a stimulation of gene targeting efficiency by delivery of nuclease activity during AMT. The first approach was based on translocation of T-strands piloted by a chimerical NLS-VirD2-I-SceI-VirF^CT^ protein using an *Agrobacterium* strain with a *virD2* deletion (LBA2556), the second one on T-strand delivery via an isogenic *Agrobacterium* strain containing a WT *VirD2* locus (LBA1100) concomitant with transfer of NLS-I-SceI-VirF^CT^. Apart from the plasmids expressing the proteins of interest, both strains contained the binary vector p14-2GKX (pSDM14:*rightborder:UAS7*: *KanMX*:*UAS10:leftborder*). T-DNA derived from p14-2GKX can integrate into the yeast genome via the HR pathway at the target locus consisting of *UAS7*:*I-SceI RS*:*URA3*:*I-SceI RS*:*UAS10*. The translocated I-SceI fusion proteins might enhance this by inducing the formation of DSBs at the target locus.

**Figure 4:**
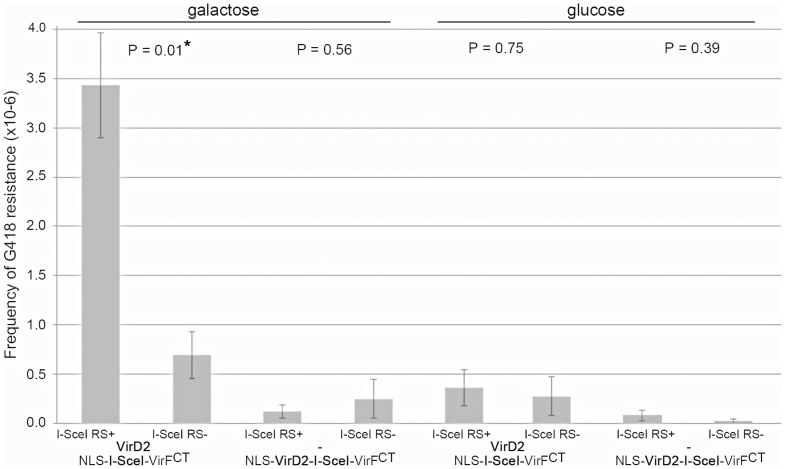
Influence of galactose or glucose treatment on the I-SceI assisted integration of T-DNA by HR. The yeast strain RSY12 was cocultivated with LBA1100 harboring p14-2GKX (pSDM14:*rightborder:UAS7*: *KanMX*:*UAS10:leftborder*) and pBFF I-SceI or LBA2556 p14-2GKX and pBFF VirD2-I-SceI. Different yeast strains with and without I SceI recognition sites between the uracil autotrophy marker and the *UAS* promoter sequences making up the target locus (Figure 2A, 2C) were compared, here indicated as I-Sce RS^+^ and I-SceI RS^−^. A horizontal line is used to mark the presence of galactose (inducing nucleosome removal *UAS* regions at the target locus) or glucose (inducing nucleosome formation). Every bar represents that average of four independent experiments (n = 4). The p-values were calculated performing a two-tailed heteroscedastic Student's T-test. Error bars indicate the SEM. The asterisk indicates statistical significance with α = 0.05. The transformation frequencies were calculated by dividing the total number of G418 resistant colonies by an estimate of the total number of yeast cells.

**Figure 5:**
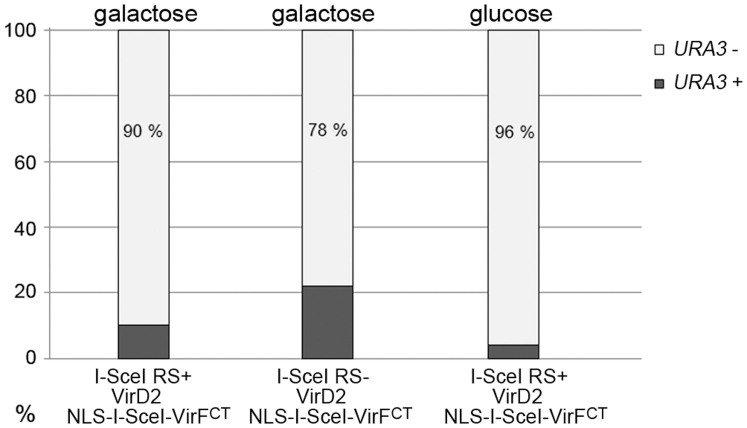
Assay on uracil auxotrophy of G418 resistant yeast transformants. As a consequence of the integration of the T-DNA (*UAS10*:*kanMX:UAS7*) mediated by HR, the uracil autotrophy marker of the target locus can be replaced by the KanMX marker gene leading to Ura auxotrophy (here indicated as *URA3*−). The off-target integration of the T-DNA leaves the *URA3* gene intact (here indicated as *URA3*+). Each bar represents the average of fifty G418 resistant colonies assayed for growth on SD-ura.

The observed AMT frequencies are depicted in Figure 4. As can be seen in this figure, the usage of a yeast strain harboring a target locus with two I-SceI restriction sites flanking the *URA3* marker gene did not lead to an increase in transformation efficiency using LBA2556 harboring p14-2GKX (which delivers T-DNA with *UAS7*: *KanMX*:*UAS10*) and pBFF VirD2-I-SceI. A different result was obtained when these yeast cells were cocultivated with LBA1100 harboring p14-2GKX and pBFF I-SceI. Here, WT VirD2 directed the translocation of the T-strand while NLS-I-SceI-VirFCT was translocated separately to induce DSBs at the target locus. Now a significantly higher transformation efficiency was obtained when I-SceI recognition were present sites at the target locus (Figure 5, first two bars).

Interestingly, this positive effect of translocated NLS-I-SceI-VirF^CT^ on T-DNA integration was only found when galactose had been present during the cocultivation period but not when glucose was added to the induction medium instead. This observation suggests that the I-SceI nuclease gains much better access to its recognition sites in the genome if nucleosomes are absent due to cultivation on galactose medium. The formation of DSBs in turn enhances T-DNA integration at the target locus. Reversely, addition of glucose apparently leads to reduced accessibility of the target locus for the I-SceI fusion protein.

To verify that the majority of the integration events indeed took place at the target locus, G418 resistant colonies were assessed for loss of uracil autotrophy. As shown for colonies resulting from three different cultivations (Figure 5), the majority of these cells had lost the *URA3* marker, indicative of HR-mediated gene replacement. The few colonies that retained uracil autotrophy possibly represent single cross-overs at the target locus or at native *UAS7* or *UAS10* promoter sequences that are part of the cognate *GAL* genes.

## Discussion

Using a yeast model system, we investigated possible effects of different chromatin states on gene targeting by means of *Agrobacterium* mediated transformation (AMT). We also assessed the contribution of co-delivered fusion proteins containing I-SceI nuclease domains to facilitate targeted integration of T-DNA during the AMT. Based on our findings, we conclude that enhancing or decreasing nucleosome occupancy of a target locus has no significant impact on the integration efficiency in the absence of a site specific nuclease. For T-DNA integration, the presence of nucleosomes might not be limiting because T-DNA tends to integrate at sites where the genomic DNA is broken. In these situations the DNA repair mechanisms are likely to remove the nucleosomes during the process of DNA repair. However, T-DNA integration at intended target sites was clearly enhanced by simultaneous transfer of I-SceI nuclease to galactose-grown yeast cells. This effect might very well be attributed to galactose-induced removal of nucleosomes from the nuclease target sites, thus making them more accessible for the nuclease to induce the double stranded breaks (DSBs) that are required for enhancement of the T-DNA integration efficiency. However, it cannot be excluded that other changes occurring during growth on galactose containing medium might play a role as well. In any case, the obtained results clearly demonstrated that the combined translocation of nuclease proteins and T-strands during AMT can facilitate HR mediated integration of T-DNA.

After evaluating the performance of the experimental tools that were generated, experiments were performed to determine if the nucleosome occupancy of a target locus would have an impact on the efficiency of T-DNA integration via the HR pathway. As described above, we used promoter sequences from genes that are part of the yeast galactose pathway as the regions homologous to the incoming T-strand at the target locus. The nucleosome occupancy of the *UAS* type promoter sequences used has been well characterized. For *UAS10* it has been shown that galactose-induced association of the transcriptional activator Gal4, rapidly recruits the nucleosome remodeling complex SWI/SNF which then removes nucleosomes from this promoter^26^. In another study, a Gal4 binding site was transferred to a histidine promoter located on a yeast episome^40^. On galactose-induced expression of Gal4, nucleosomes adjacent to the Gal4 binding site were removed. These findings demonstrate that Gal4-mediated nucleosome removal was independent of the sequences flanking the Gal4 binding site. Concerning the studies mentioned above, it should be safe to assume that the unaltered *UAS* promoters used still exhibit the reported galactose- and glucose dependent regulation of nucleosome occupancy.

Our experiments showed that HR-mediated integration of T-DNA at the target locus was independent of the presence or absence of nucleosomes or other effects that a change of carbon source might have. This is an intriguing observation since for yeast cells transformed with linear dsDNA using a LiAc/ssDNA/PEG transformation protocol, the presence of nucleosomes at the genomic locus carrying sequence homology to the incoming DNA fragment is known to negatively affect the transformation efficiency^41^. Our findings however are in line with earlier research showing that integrated T-DNAs are not enriched at genomic sites that have a methylation status indicative for transcriptional activity^10^.

The results regarding the effects of nuclease proteins translocated concomitantly with T-strands into yeast cell showed that of the proteins tested, translocation of NLS-I-SceI-VirF^CT^ clearly enhanced T-DNA integration, but only when I-SceI recognition sites were present at the target locus (Figure 4). The majority of the integration events were correctly targeted and the presence of I-SceI recognition sites at the target locus even led to a slight increase in correctly targeted integration events (Figure 5). These data provide evidence that it is indeed feasible to translocate nucleases simultaneously with T-strands via the T4SS of *Agrobacterium* to introduce DSBs in the host genome during in order to enhance gene targeting. The improvements regarding gene targeting perhaps remained relatively modest because a yeast model was used that already has a very effective HR pathway. However, in systems where the HR mechanism is much less proficient, delivery of targetable nuclease proteins might very well serve to improve gene targeting. Procedures used thus far to improve T-DNA targeting, have always relied on the introduction of gene constructs encoding nucleases of interest, but our methodology of “protein therapy” in combination with a gene targeting construct has the advantage that no extra nucleic acids need to be introduced besides the gene targeting construct.

Strikingly, as can be observed in Figure 4, the enhanced transformation efficiency that was obtained by the *Agrobacterium* mediated translocation of NLS-I-SceI-VirF^CT^ was abolished if glucose was present in the medium instead of galactose. As is illustrated in Figure 3, the presence of galactose or glucose had no effect on the transformation efficiency when the T-DNA was homologous to the *PDC6* locus. Since the *PDC6* locus is not part of the galactose pathway and the nucleosome occupancy should therefore not be affected by the carbon source, this experiment excluded a general effect of the utilized carbon source on the T-DNA integration frequency. Therefore, although additional changes might have occurred, repression of AMT frequency in the presence of glucose is likely to be indicative of glucose induced nucleosome formation at the *UAS* target sites, thereby reducing the availability of the I-SceI binding sites for digestion by NLS-I-SceI-VirF^CT^. The consequent reduction in DSB formation at the target locus would then account for the reduced transformation efficiency.

While an *in vivo* assay demonstrated that NLS-VirD2-I-SceI-VirF^CT^ still possessed nuclease activity, attempts to use this chimerical protein for T-strand delivery as well as DSB induction resulted in very low transformation efficiencies, yielding too few yeast transformants to give a reliable estimate of the T-DNA targeting efficiency (Figure 4). The observed low transformation efficiency could be due to reduced T4SS mediated translocation of NLS-VirD2-I-SceI-VirF^CT^ compared to WT VirD2 or to reduced relaxase activity of VirD2 when fused to other protein moieties. Both issues would lead to less T-strands being transferred, resulting into lower transformation efficiencies. However, the chimerical VirD2 proteins can still support enzyme activity for DNA metabolism as shown for the ability of NLS-VirD2-I-Cre-VirFCT to support *in vivo* Cre-mediated recombination on a yeast target locus. In parallel experiments performed in plant cells, it was shown that also NLS-VirD2-I-SceI-VirFCT can be transferred during AMT, while retaining nuclease activity^23^. Therefore, unless bottlenecks can be adequately addressed, the collected data indicate that the most effective approach to transfer nuclease proteins to induce DSBs concomitant with AMT is the separate translocation of the nuclease proteins rather than constructing more complex VirD2 fusion proteins. When only assessing the transfer of a very similar NLS-Cre-VirFCT protein to yeast cells containing a *lox*P flanked locus, it was previously found that up to 1% of yeast target cells exhibited the recombined genotype after AMT^22^.

In summary, the data obtained using the yeast model system for AMT indicated that *Agrobacterium* mediated translocation of the homing endonuclease I-SceI has a positive effect on the integration of T-DNA via the homologous repair (HR) pathway. We found no evidence that the nucleosome occupancy status of the future integration site had an impact on the transformation efficiency. However, we did observe that the presence of glucose, that is known to lead to an elevation of the nucleosome occupancy of the *UAS* sequences present at the target locus, removed the positive effect of *Agrobacterium* mediated transfer of specific nucleases on T-DNA integration at the target locus. For an optimal contribution of DSB induction by specific nucleases to gene targeting, it is apparently pivotal to take the nucleosome occupancy of the intended target site into account.

## Figures and Tables

**Table 1:** Oligos designed for the amplification of the galactose pathway promoter sequences *UAS7* and *UAS10* and the *URA3* marker gene

Oligo name	Nucleotide sequence (5′-3′)
UASGAL7TTGAL10 *Bam*HI F	GAGTAGGATCCAATATTCAACTGTTTTTTTTTATCATG
UASGAL7TTGAL10 *Xho*I R	ATCGCTCGAGTTGCCAGCTTACTATCCTTC
UASGAL10-GAL1 *Spe*I F	TGACACTAGTTTGAATTTTCAAAAATTCTTACTTTTTTTTTGG
UASGAL10-GAL1 *Bam*HI R	GCATGGATCCTTGACGTTAAAGTATAGAGGT
URA Xho FW	TCCTCTCGAGCTTTTCAATTCAATTCASTC
URA Spe RV	TGGCACTAGTGGGTAATAACTGATATAATTAAATT

**Table 2:** Oligos used for the construction of DNA fragments added to the integrative vector pID2GU (pINT:*PDC6*:*UAS10*:*URA3*:*UAS7*:*PDC6*) resulting in pID2G2SU equipped with I *Sce*I recognition sites (pINT:*PDC6*:*UAS10*:*I-SceI RS*:*URA3*:*I-SceI RS*:*UAS7*:*PDC6*) and pID2G2LU equipped with Lox sites (pINT:*PDC6*:*UAS10*:*lox*P:*URA3*:*lox*P:*UAS7*:*PDC6*)

Plasmid	Oligo name	Nucleotide sequence (5′-3′)
pID2G2SU	scesalxhoFW scesalxhoRV scespexbaFW scespexbaRV	TCGACCTTAAGTTACGCTAGGGATAACAGGGTAATATAAC TCGAGTTATATTACCCTGTTATCCCTAGCGTAACTTAAGG CTAGTACGCTAGGGATAACAGGGTAATATAACGGAATTCT CTAGAGAATTCCGTTATATTACCCTGTTATCCCTAGCGTA
pID2G2LU	loxsalxhoFW loxsalxhoRV loxspexbaFW loxspexbaRV	TCGACATAACTTCGTATAGCATACATTATACGAAGTTATC TCGAGATAACTTCGTATAATGTATGCTATACGAAGTTATG CTAGTATAACTTCGTATAGCATACATTATACGAAGTTATT CTAGAATAACTTCGTATAATGTATGCTATACGAAGTTATA

**Table 3:** Oligos designed for the amplification of the *PDC6* locus and the *KanMX* marker gene. Restriction sites added to the PCR primer sequences are underlined

Oligo name	Nucleotide sequence (5′-3′)
*PDC6*FW	TAATAGATCTAGGTAAATAAATGTGCAGATGCA
*PDC6*RV	TAATAGATCTTGAACAAAGGGCGAAACTTCGCA
Kan Xho	TAATCTCGAGTTTAGCTTGCCTCGTC
Kan Spe	TAATACTAGTTTTCGACACTGGATGG

**Table 4:** Overview of all the constructs mentioned in the experimental setup

Abbreviation	Name	Description	Vector type, organism
pIDU	pSDM3585	pINT:*PDC6*:***URA3****:PDC6*	Integrative vector, yeast
pID2GU	pSDM3588	pINT:*PDC6*:*UAS10*:***URA3***:*UAS7*:*PDC6*	Integrative vector, yeast
pID2G2LU	pSDM3586	pINT:*PDC6*:*UAS10*:*lox*P:***URA3***:*lox*P:*UAS7*:*PDC6*	Integrative vector, yeast
pID2G2SU	pSDM3587	pINT:*PDC6*:*UAS10*:*I-SceI RS*:*URA3*:*I-SceI RS*:*UAS7*:*PDC6*	Integrative vector, yeast
pRS425 VirD2-I-SceI	pSDM3592	pRS425:p*ADH:****NLS*::*virD2*::*I-SceI*::*virF^CT^***	Expression vector, yeast
pRS425 VirD2-Cre	pSDM3591	pRS425:p*ADH:****FLAG*::*NLS*::*virD2*::*Cre*::*virF^CT^***	Expression vector, yeast
pBFF I-SceI	pSDM8017	pBFF:***FLAG*::*NLS*::*I-SceI*::*virF^CT^***	Expression vector, *Agrobacterium*
pBFF VirD2-I-SceI	pSDM3884	pBFF:***FLAG*::*NLS*::*virD2*::*I-SceI*::*virF^CT^***	Expression vector, *Agrobacterium*
pBFF Cre	pSDM8019	pBFF:***FLAG*::*NLS*::*Cre*::*virF^CT^***	Expression vector, *Agrobacterium*
pBFF VirD2-Cre	pSDM8020	pBFF:***FLAG*::*NLS*::*virD2*::*Cre*::*virF^CT^***	Expression vector, *Agrobacterium*
p14-PDC6KX	pSDM3590	pSDM14:*rightborder*:*PDC6*:***KanMX****:PDC6:leftborder*	Binary vector, *Agrobacterium*
P14-2GKX	pSDM3589	pSDM14:*rightborder:UAS7*: ***KanMX***:*UAS10:leftborder*	Binary vector, *Agrobacterium*
	pSDM8000	pSDM14:*rightborder:****KanMX****:leftborder*^16^	Binary vector, *Agrobacterium*

For convenience, the constructs were given a short working name. All uninterrupted ORFs in the description are depicted in bold writing. The abbreviation “I-SceI-RS” indicates recognition sites for I-SceI.
